# Histopathological Analysis of the Effect of Photodynamic Action on Post-Chemotherapy Excised Breast Cancer Tissue

**DOI:** 10.3390/medicina58060700

**Published:** 2022-05-25

**Authors:** Elżbieta Ostańska, Edyta Barnaś, Dorota Bartusik-Aebisher, Klaudia Dynarowicz, Magdalena Szpunar, Joanna Skręt-Magierło, David Aebisher

**Affiliations:** 1 Clinical Department of Pathology, Frederick Chopin Clinical Provincial Hospital No. 1, 35-055 Rzeszów, Poland; elaostanska@gmail.com; 2 Department of Midwifery, Medical College of the University of Rzeszów, University of Rzeszów, 35-959 Rzeszów, Poland; ebarnas@interia.eu (E.B.); joannaskret@wp.pl (J.S.-M.); 3 Department of Biochemistry and General Chemistry, Medical College of the University of Rzeszów, University of Rzeszów, 35-959 Rzeszów, Poland; dbartusikaebisher@ur.edu.pl; 4 Center for Innovative Research in Medical and Natural Sciences, Medical College of the University of Rzeszów, University of Rzeszów, 35-310 Rzeszów, Poland; kdynarowicz@ur.edu.pl; 5 Students English Division Science Club, Medical College of the University of Rzeszów, 35-959 Rzeszów, Poland; szpunarmag@gmail.com; 6 Department of Photomedicine and Physical Chemistry, Medical College of the University of Rzeszów, University of Rzeszów, 35-959 Rzeszów, Poland

**Keywords:** photodynamic therapy, histology, breast cancer, photosensitizer

## Abstract

*Background and objectives*: Breast cancer is the most commonly diagnosed cancer in women and its mortality is increasing. Therefore, research to improve treatment is of paramount importance. One method of treatment is photodynamic therapy. Photodynamic therapy selectively stimulates apoptosis in photosensitizer-treated neoplastic breast cells as a result of cytotoxic singlet oxygen generation via collisions between triplet excited state photosensitizer and triplet ground state oxygen upon tissue irradiation. The aim of this study was to evaluate the effects of photodynamic action on cancerous breast tissue samples as a model of photodynamic therapy. *Materials and Methods*: Breast cancer tissue samples were obtained from post-operative material and the patterns of histopathological changes in breast cancer tissue before and after photodynamic action on post-chemotherapy tissue were evaluated. Excised tissue samples were obtained from 48 female breast cancer patients who had previously undergone chemotherapy. Breast cancer tissues for this study were taken from macroscopically visible tumors larger than 10 mm. Histopathological analysis was performed to evaluate any morphological changes prior to and after photodynamic action on the post-chemotherapy tissue samples. Eighteen breast cancer tissue samples were analyzed before chemotherapy, fifteen after chemotherapy, and fifteen samples were analyzed after chemotherapy and application of photodynamic action. The photosensitizer Rose Bengal was applied to the samples subjected to photodynamic action. *Results*: Photodynamic action on post-chemotherapy neoplastic tissue showed histological changes under a light microscope. The results showed that morphological changes in breast cancer tissues after chemotherapy and photodynamic action were dependent on the concentration of Rose Bengal. In all cases, follow-up imaging showed tumor shrinkage of an average of 35% from baseline size. *Conclusions*: Histopathological examination revealed photosensitizer-concentration-dependent changes after photodynamic action in excised post-chemotherapy tissue. The effects of photodynamic action observed in this study suggest that the application of photodynamic therapy after chemotherapy can aid in breast cancer cell eradication.

## 1. Introduction

Breast cancer is the most commonly diagnosed cancer with approximately 2.3 million new cases diagnosed in 2020 [1]. The main treatments for breast cancer are surgery [2], chemotherapy [3], radiation therapy [4], hormone therapy [5], and targeted therapy that uses drugs to target specific genes [6] and proteins [7] that are involved in the growth and survival of breast cancer cells. In recent years, photodynamic therapy (PDT) has been an interesting therapeutic approach in the treatment of cancer [8]. Currently, published studies show that PDT in combination with chemotherapy has become an available cancer treatment which uses functionalized organic nanoparticles (NPs) for effective PDT treatment of breast cancer [9]. One more possible solution to increase effectiveness for PDT in breast cancer could be the use of gold nanoparticles (AuNPs) in combination with cannabidiol (CBD), a Cannabis derivative from the Cannabis sativa; this has also been reported to be effective against breast cancer cell lines [10]. Both of the aforementioned examples have highlighted targeted drug delivery systems that improve the biodistribution of photosensitizers (PSs) in breast cancer tumors. The advantages that arise from a combination of PDT and chemotherapy are (1) high selectivity and (2) a decrease in breast cancer drug resistance. Due to metastases and recurrences, complete elimination of breast cancer is a significant challenge. Modern oncological research shows that cancer is not a simple local disease, but a local manifestation of a systemic disease. Current cancer treatments make it difficult to remove all the cancer cells from the body. Traditional strategies, including chemotherapy and radiotherapy, often lead to the development of cancer cell resistance to treatment [1,11]. Consequently, tumor relapse and metastasis are inevitable after treatment. Combining chemotherapy with photodynamic action offers the possibility of an effective treatment of cancer and an improvement in the living conditions of patients suffering from it. The main aim of this experiment was to histologically evaluate ex vivo breast cancer tumor tissue sections that were treated with several Rose Bengal concentrations and irradiated with visible light to incite photodynamic action. Histological analysis is the gold standard for tissue examination and this methodology is useful for diagnostics, and for monitoring the presence, distribution and accumulations of cellular degradation and accumulations of cellular degradation products arising from tissue treatment [12].

## 2. Materials and Methods

All studies were performed under the approval (No. 10/11/2018) of the Bioethics Committee at the University of Rzeszów. The study was conducted from March 2019 to December 2020. Patients were selected for the study with core needle biopsy and Vacuum-Assisted Biopsy clinical stage IIB and III (locally advanced and metastatic breast cancer) diagnosed breast cancer in whom, based on imaging tests and immunohistochemical examination of predictive factors (ER, PR, Ki67, HER2), cycles of neoadjuvant chemotherapy were implemented. These included 4 cycles of the AC regimen (ADM + CTX) (Adriamycin + Cyclophosphamide) administered every 3 weeks and 10–12 doses of Paclitaxel. In two HER2 (+) patients, 5 doses of Herceptin were administered. Approximately 6 months after the start of treatment, the patients underwent routine mastectomy surgery at the Department of General and Oncological Surgery, Provincial Hospital No. 1 in Rzeszów. Breast surgical tissues were delivered to the Department of Pathomorphology. All 48 female breast cancer tissue samples were divided into three groups. The first group of samples were breast cancer tissues prior to chemotherapy (*n* = 18), the second group (*n* = 15) were breast cancer tissues after chemotherapy that were subjected to a histopathological analysis, and the third breast cancer tissues group (*n* = 15) were post-chemotherapy samples that were used for the application of photodynamic action with Rose Bengal (RB) prior to histopathological analysis. Rose Bengal concentrations were 0.02 g/mL (5 samples), 0.03 g/mL (5 samples), and 0.05 g/mL (5 samples). Breast cancer was excised from solid tumors larger than 10 mm in diameter and stored in a BioBank at −78 °C prior to use.

Post-operative breast cancer tissue samples were fixed for 24 h in 10% buffered formalin. Then, specimens were routinely taken for further histopathological examinations. Sections were left in 10% buffered formalin until the next day for further fixation. The next steps during histopathological procedures were (1) formalin decanting, (2) rinsing in running water (3) further fixation in 70% alcohol, (4) preparation in a Leica TP 1020 tissue processor with ethyl alcohols, xylene and paraffin, (5) production of paraffin blocks using the AP280 sealer, (6) cutting tissue sections from blocks using Rotary microtome RM2245, (7) Hematoxylin–Eosin (H + E) routine staining using the Leica ST5020, and (8) securing sections on slides using the Leica SV5030 cap.

Hematoxylin+Eosin is the most common staining in which the cytoplasm of cells is shown in pink and the nucleus in blue. Routine histopathological diagnosis was performed with a Leica DM1000 LED light microscope. Fifteen breast tissue samples were selected for photodynamic action based on routine histopathological examinations.

Rose Bengal disodium salt (95%) was purchased from Sigma-Aldrich and used as received. Water used for preparation of Rose Bengal stock solution was purified with an AquaB Duo reverse osmosis water treatment system, Fresenius Medical Care, Singapore Pte. Ltd. Rose Bengal was applied at concentrations of 0.02 g/mL, 0.03 g/mL and 0.05 g/mL. Table 1 summarizes the concentration of Rose Bengal applied to the excised tissues.

Once received from the Pathomorphological Department, the breast cancer tissues were stored in 15 mL of deionized water in 15 mL polypropylene graduated conical test tubes fitted with a screw tight cap (Kartell Labware, Milano, Italy) at 5 °C. The dimensions of the breast cancer tissues were approximately 6 mm × 4 mm × 3 mm. A 10 mL syringe and an injection needle (0.8 mm) were used to deliver Rose Bengal solution to the breast cancer tissue. Photodynamic action was initiated by irradiation in 5 separate trials for each Rose Bengal concentration (0.02 g/mL, 0.03 g/mL, and 0.05 g/mL).

Before tissue illumination, each sample was covered with 0.1 mL RB. This amount of RB allowed for complete coverage of the examined tissues. Subsequently, the tissues were covered and kept from the light for 30 min at 18 °C to allow the Rose Bengal to penetrate into the tissue. For eliciting photodynamic action, a solid-state laser (LD Pumped All-Solid-State Green Laser, MGL-III-532 nm/300 mW) coupled to a fiber optic cable was used to deliver 532 nm light to the treated tissue samples for 15 min. The light cone covered the entire tumor and was distributed uniformly on the tissue surface. In addition, the distance of the light source from the tissue surface was selected so as not to cause excessive heating or drying of the tissue. The temperature on the surface of the tissue after 15 min of exposure did not exceed 30 °C. The radiant power of the 532 nm light was measured with a Newport power meter model 1918-C.

In the next step, irradiated tissue was then fixed in 10% buffered formalin, and subjected to histopathological preparation. Tissue sample images before treatment, after chemotherapy, after chemotherapy and photodynamic action were compared using light microscopy (Leica DM1000 LED).

## 3. Results

We evaluated the differences in tissue by comparing microscopic images before chemotherapy, after chemotherapy and after chemotherapy and photodynamic action. The results showed that morphological changes of breast cancer tissues after chemotherapy and photodynamic action were dependent on the concentration of Rose Bengal.

As the concentration of Rose Bengal increased, chromatin condensation of cancer cells became more apparent and, at the same time cell, nuclei pyknosis (irreversible chromatin condensation in the nucleus of a cell undergoing necrosis or apoptosis) was enhanced. In histopathological studies of the 18 breast cancer tissue samples before chemotherapy, images showed loss of healthy architecture and increased number of tumor cells. In the 15 samples of breast cancer tissue after chemotherapy, a change in histological differentiation was seen.

**Figure 1:**
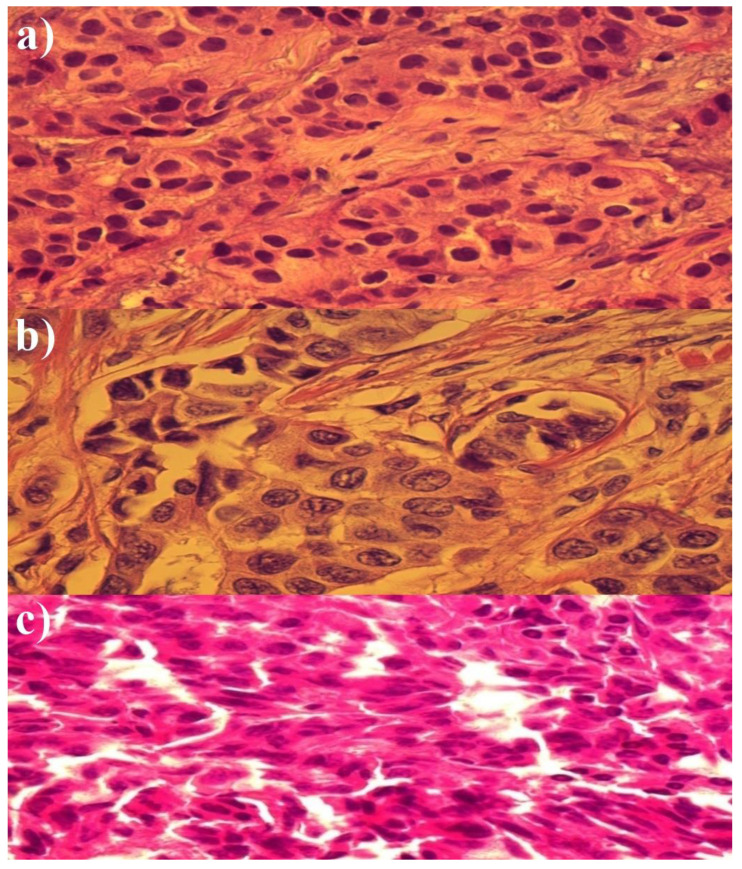
Image of histopathological material of cancer tissue (**a**) before chemotherapy and PDT, (**b**) after chemotherapy, (**c**) after chemotherapy and photodynamic action (Rose Bengal 0.02 g/mL).

The histopathological changes after chemotherapy varied between patients. In 15 breast cancer samples after chemotherapy, changes involving the enlargement and contraction of the nucleus and neoplastic cells are visible. In all cases, follow-up imaging showed tumor shrinkage of an average of 35% from baseline size. Figure 1 shows representative histopathological images of breast cancer tissue: (a) before chemotherapy, (b) after chemotherapy, and (c) after chemotherapy and photodynamic action.

Figure 1b (after chemotherapy) shows gland-shaped abnormalities. Additionally, edema and increased eosinophilic character of the cytoplasm were observed. Moreover, some cancer cells have aquatic lesions and nuclear pyknosis. In turn, Figure 1c (after chemotherapy and photodynamic action) shows more advanced destruction of cancer glands. Most of the cells were found to be ruptured. In addition, the cytoplasm became homogeneous and eosinophilic with increased pyknosis and deformation of the cell nuclei.

**Figure 2:**
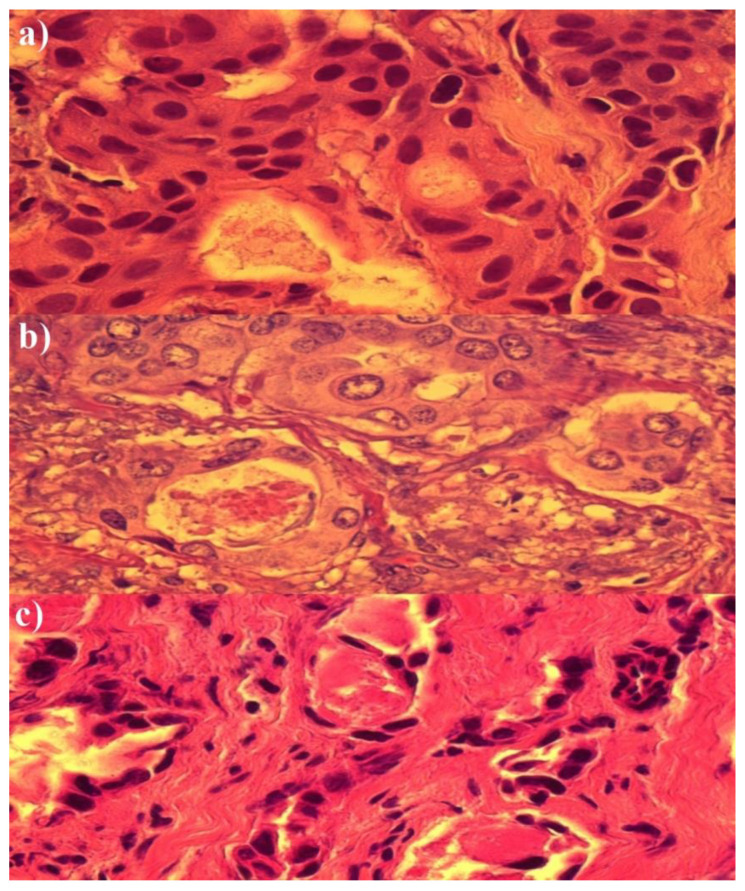
The representative image of histopathological material of cancer tissue (**a**) before chemotherapy, (**b**) after chemotherapy, and (**c**) after chemotherapy and photodynamic action (Rose Bengal 0.03 g/mL).

Figure 2 shows histopathological images of breast cancer tissue: (a) before chemotherapy, (b) after chemotherapy, and (c) after chemotherapy and photodynamic action (Rose Bengal 0.03 g/mL).

Figure 2b (after chemotherapy) presents slight changes in the form of cytoplasmic edema, with the formation of aquatic lesions. Cells merge, and the cell membrane is lost. There are slight changes in the nuclei. In turn, Figure 2c (after chemotherapy and photodynamic action) shows complete visible degradation of the cytoplasm, nuclei with bizarre blotchy shapes, and chromatin homogenization.

**Figure 3:**
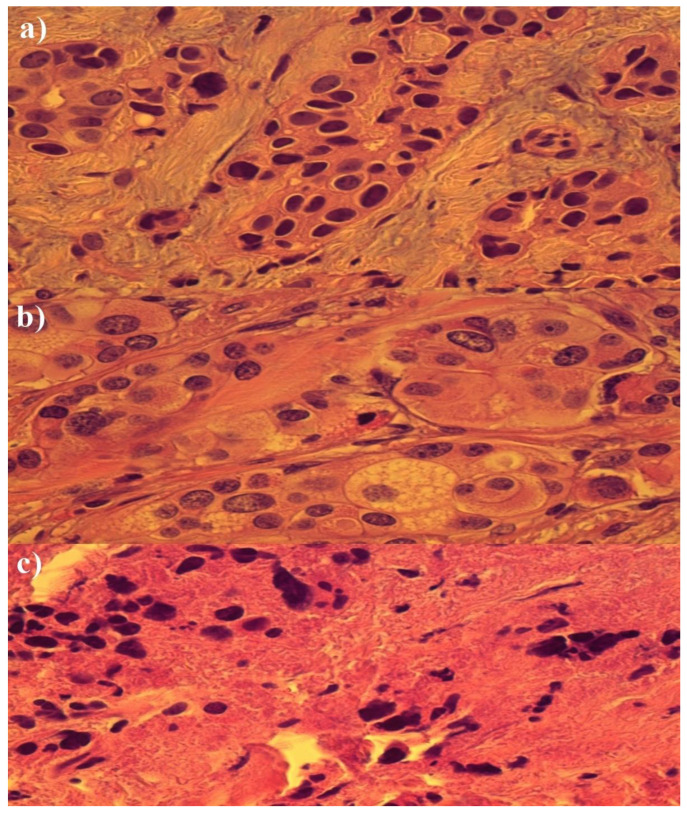
Histopathological image of breast cancer tissue, (**a**) before chemotherapy, (**b**) after chemotherapy, (**c**) after chemotherapy and photodynamic action.

Figure 3b (after chemotherapy) shows current condensation of nuclear chromatin in a few cancer cells. In turn, Figure 3c (after chemotherapy and photodynamic action) presents visible homogenization of the cytoplasm with the disappearance of cytoplasmic membranes, and degradation of nuclear chromatin with the presence of a few changes similar to apoptosis. Figure 3b (after chemotherapy) shows advanced aquatic changes in the cytoplasm and nuclear pyknosis. In turn, Figure 3c (after chemotherapy and photodynamic action) shows the disappearance of cytoplasmic membranes, further intensification of pyknosis, partially complete homogenization and chromatin breakdown.

In this study, we used a Rose Bengal solution with concentrations of 0.02 g/mL, 0.03 g/mL, and 0.05 g/mL. We used each concentration five times in post-operative breast cancer tissue samples after chemotherapy and performed an *in vitro* photodynamic therapy modeling experiment. To describe the results of the histopathological examination, we chose an area in which 100 cells were visible. We counted all cells in the area (100 cells), and then, on the basis of their condition, we counted the fraction of cells that lost vitality and were agreed to be dead (Table 2). The described analytical method is a common procedure for the statistical analysis of histological preparations used in pathomorphology.

As the concentration increased, the number of dead cells increased. The application of 0.05 g/mL of Rose Bengal solution showed the highest number of killed cells (93 ± 3%) when compared to 0.03 g/mL and 0.02 g/mL. During all experiments, the 532 nm light covered the entire area of breast cancer tissue. The temperature on the surface of the tissue was room temperature, and the tissue did not dry out the tissue during the course of the experiment. The irradiation of samples for 15 min turned out to be sufficient for the detection of damage caused by photodynamic action *in vitro*. Lower concentrations of Rose Bengal at 0.02 g/mL and 0.03 g/mL resulted in 32 ± 2% and 46 ± 3% killing effect, respectively.

The optimization results are presented in Table 3, which shows that the best results were obtained when the breast tissue was placed 15 cm from the laser light source and exposed to radiation for 15 min. In this setup, the 532 nm light covered the top tissue surface.

## 4. Discussion

The destruction of tumors by PDT is a phenomenon that has been known for about a century [13]. The effectiveness of PDT significantly depends on the PS dose, type, and cellular localization of the PS; the intensity, duration and wavelength of light; as well as the availability of oxygen at the target site [14]. In our experiment, potential cellular targets for PDT were studied. All cancer tissues after chemotherapy showed various degrees of cell changes, mainly consisting of the swelling of cancer cells, the formation of aquatic changes in the cytoplasm (Figure 1b, Figure 2b and Figure 3b), and changes in the cell nuclei showing chromatin homogenization. The same tissues after photodynamic action showed further changes in the degradation of the neoplastic tissue (a strength of the experiment). These changes showed a different degree of intensity, from small changes consisting of the condensation of nuclear chromatin, the formation of blotchy and bizarre (bizarre) forms, with assuming forms similar to the image of apoptosis in tissues *in vivo* and with a tendency of cytoplasm decay, to complete cell degradation, with the formation of amorphous homogeneous weaving. These changes showed varying degrees of intensity even within one cross-section of the tumor tissue. The intensity of the changes was not directly proportional to the concentration of the photosensitizer in all cases. The power and density of the 532 nm light delivered to the tissue were also important. Besides photodynamic cell killing, indirect effects were possible, due to the possibility of incorporating the Rose Bengal into the cellular membranes. The products of photodynamic action are reactive oxygen species (ROS) such as singlet oxygen that are capable of destroying cancer cells. Their activity directly causes the death of neoplastic cells through necrosis or apoptosis, destruction of tumor blood vessels, and stimulation of the immune system [15]. The main limitation of the study was the method of application of photosensitizers and their histopathological evaluation after the irradiation.

Before starting our research, a literature review was carried out to become acquainted with the experimental procedures of other research groups, which generates interest in taking up the topic of oncology therapy in laboratory. A number of studies have addressed the possible involvement of DNA damage in PDT phototoxicity combined with chemotherapy. The combination of chemotherapy with PDT enables precise drug delivery and continuous control of its release [16,17]. Additionally, light is the main factor in stimulating (inducing) the photodynamic response.

After administration of PS into patients, exposure to natural and artificial light must be limited due to the possibility of damaging the healthy cells [15,18,19]. One method of delivering light to deeper tissues in PDT therapy is with the use of up-conversion nanoparticles (UCNPs) that absorb near-infrared light and emit visible light.

**Figure 4:**
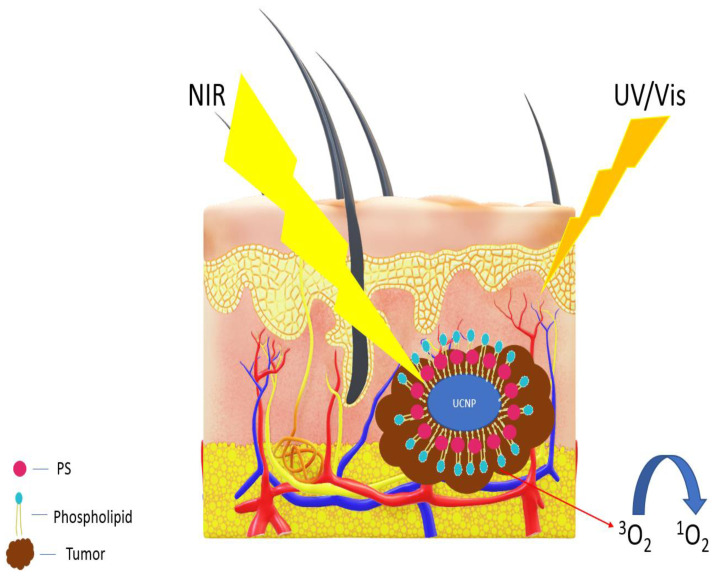
Photodynamic therapy (PDT) with UCNP using near-infrared (NIR) light.

Over the past few years, many research groups have demonstrated the effectiveness of UCNP-based PDT both *in vitro* and *in vivo* [20,21]. The use of UCNP is a leading topic of many research groups in Europe and in the world [20]. Figure 4 shows a simplified diagram of the operation of the PDT with UNCP.

### PDT-Chemotherapy Nanoparticles

Chemotherapy has been at the forefront of cancer treatment for several decades, yet other less harsh methods have been sought. Photodynamic therapy offers the possibility of ‘defeating’ cancer by partially or completely destroying cancer cells [22,23,24]. Over the last twenty years, research and review studies have demonstrated the effectiveness of this method. However, in order to precisely and permanently cure the tumor, it is worth using an adjunct therapy such as PDT in combination with chemotherapy. The purpose of this is to achieve an effective ability to exert maximum antitumor capacity and inspire antitumor immunity to prevent tumor relapse and metastasis.

In a study by Jin et al., UCNP was prepared in a shell of micelles that generated ROS. The applied complex worked in conjunction with an anticancer chemotherapeutic agent, which is an example of a positive correlation between PDT and chemotherapy. Both PDT agents and chemotherapy can activate antitumor immunity by inducing immunogenic cell death. Experiments have shown that intravenous administration of multifunctional nanocarriers in combination with non-invasive NIR irradiation destroys orthotopic tumors and effectively inhibits metastasis to other tissues and organs. This study confirms the effectiveness of chemo-photodynamic therapy in combination with antitumor immunity in the treatment of metastatic cancer [25].

According to Huang et al., the treatment of bone metastases, for example, is associated with certain limitations. Among them are the lack of selectivity of the applied therapies, severe toxicity and low efficiency. The synergy of chemotherapy with PDT may be of key importance to the achievements of existing methods [26]. Jiang et al., in order to improve the effectiveness of PDT, used PS and doxorubicin (DOX), which were coated with polyethylene glycol. The complex was found to have high drug loading capacity for both PS and the chemotherapeutic agent, allowing the combination of chemotherapeutic treatment of cancer with simultaneous deep penetration and accumulation of the drug in the internal tumor regions. The experiment confirmed the potential of the nanocomplex and its potential development directions [27]. Wang et al. constructed nanocomposites based on a magnetic nanoparticle to deliver chlorin (Ce6) and the therapeutic drug DOX. The complex resulted in tumor-targeted chemotherapy by controlled drug release and minimized side effects. The results of the experiment confirmed the highly effective antitumor properties of nanocomposites in the treatment of human tumor cells and tissues of the MCF-7 cell line. Combined chemical/photodynamic therapy significantly inhibited tumor growth in vivo [28]. Li et al. also created a nanocomplex with the photosensitizer 5-ALA and the drug DOX. Using the MCF-7 cell line, high cytotoxicity and morphological changes in cancer cells were observed. Reactive oxygen species resulting from irradiation enhanced the therapeutic effect. Uptake of the complex by cells increased apoptosis and damaged MCF-7 cells [29]. Panikar et al. presented a method of obtaining nanoliposomes targeting peptide-coupled ligands for chemo-photodynamic therapy of breast cancer. The nanocomplex contained the therapeutic drug DOX and methylene blue (MB). The energy transfer from UCNP to MB allows generation of reactive oxygen species upon activation with laser light. In the experiment, a significant decrease in cell viability was observed at the level of 95%. During chemotherapy alone and PDT alone, the levels were 77% and 84%, respectively. The obtained results confirmed the significant potential of the nanocomplex in the treatment of breast cancer [30].

Additionally, Huang et al., in their experiments, created a noncomplex comprising the Hsp90 inhibitor ganetespib (Ga) and PS such as zinc phthalocyanine (ZnPc) that accumulated in cancer cells. After exposure to laser light, the amount of ROS increased sharply. The applied Gan–ZnPc complex inhibited cell proliferation and indirectly led to apoptosis of neoplastic cells [31]. Wang et al., in their research, used a nanocomplex based on polysaccharides for delivery of the therapeutic agent DOX and 5-aminolevulinic acid (5-ALA). The platform exhibited a pH-sensitive drug release. Using a nanoplatform increased the cellular uptake of the drug and photosensitizer, which increased the effectiveness of the chemo-photodynamic therapy in MCF-7 breast cancer cells. The experiment confirmed that nanocomplexes may have enormous potential in the targeted therapy of breast cancer [32]. Hu et al., in their research, developed theranostic nanoparticles with the ability to attach DOX and PS. The aim of the research was to test the effectiveness of the therapy *in vivo* and *in vitro*. The applied complex with manganese oxide caused local hypoxia of the tumor, producing oxygen in situ. The use of the nanocomplex increased the effectiveness of pre-application PDT and enhanced the release of the drug DOX. The designed nanoparticles can be an alternative way to improve the effectiveness of both therapies, which will increase the popularity of their use [33]. Xu et al. used low molecular weight gels, an anticancer drug and PS. The results revealed that the injectable substances showed excellent antitumor efficacy due to the combination of the two treatments [34].

Liu et al. investigated the topic of theranostic nanoplatforms that combine PS and anticancer drugs. In this study, they used laponite nanoplatforms that store PS and DOX. The complex formed in a gradual and controlled manner and released the drug as a result of changes in pH and laser light intensity. Thanks to the contained substance, the complex could be used as an agent differentiating cancer cells from healthy cells (the complex was easily captured by cancer cells in an *in vivo* mouse tumor model). The experiment demonstrated higher efficiency and a stronger therapeutic effect of the therapy using the complex as opposed to single therapy. Nanoplatforms, used together with a drug and PS, are a promising methodology for highly effective chemo-phototherapy of neoplastic cells [35]. Pandya et al. developed biodegradable polymer nanoparticles containing cytotoxic drugs. Polymer nanoparticles also contained the PS tetraphenylchlorin in their structure. The research was carried out on the breast cancer cell lines MDA-MB-231 and MDA-MB-468. Higher cytotoxicity was noted in a study in which cancer cells were treated with a nanocomplex and not with the drug itself. It is worth noting that the light-induced nanoparticles had a strong effect of PDT on cancer cells. In the final stage of the experiment, it was observed that the nanoplatform can also be used as a contrast agent for cancer cells. However, this property needs to be analyzed in more detail [36]. Gaio et al. proposed a complex made of nanoparticles coated with hyaluronic acid, the drug docetaxel and PS, in order to destroy cancer cells using chemotherapy and photodynamic therapy. The mutual effects of both therapies were more effective than their single use. Only *in vitro* tests were carried out, which showed the potential of the nanocomplex in the destruction of cancer cells and the possibility of their future use under *in vivo* conditions [37]. Xu et al. developed a nanoplatform based on PEGylated gold nanorods with drug release properties. The nanoplatform was additionally equipped with DOX and a PS such as 5-ALA. The obtained complex showed excellent stability in physiological solutions. The degree of release of the contained substances depended on the pH. *In vitro* studies have shown that the nanocomplex can effectively penetrate human breast cancer cells and release the drug and PS into the cytoplasm. At the time of irradiation with near-infrared light, the production of ROS took place. A combination of chemotherapy, photodynamic therapy, and photothermal therapy may be more effective in killing breast cancer cells. The triple combination can completely inhibit tumor growth without excessive systemic toxicity. The conducted study provides hope for a complete and effective cure for cancer [38].

*In vivo* PDT causes destruction of neoplastic tissue and, to a lesser extent, the surrounding healthy tissue due to factors dependent on vascularization and oxygenation, and active processes in which the photosensitizer binds to carriers such as albumin and lipoproteins (LDL, HDL). Photodynamic therapy induces an inflammatory release of cytokines, e.g., TNFalpha. On the other hand, heat-shock proteins (Hsp) are also formed, which protect cancer cells, and macrophages may metabolize the photosensitizer. There is a question whether it is possible to modulate the tumor tissue locally in terms of its vascularization, oxygenation and inhibition of inflammatory processes just before the administration of the photosensitizer, so as to eliminate tumor protection mechanisms. The mutual correlation of PDT used after chemotherapy increases the effectiveness of cancer treatment, giving hope for a full cure for the disease.

This work shows that Rose Bengal located in a neoplastic tumor leads to the destruction of cancer tissue upon irradiation with 532 nm light. Activating Rose Bengal with laser light leads to the formation of ^1^O_2_, which at the cellular and subcellular level damage the cytoplasmic membranes, mitochondria and lysosomes. It is one of the mechanisms that causes the destruction of tumor cells.

## 5. Conclusions

Current breast cancer treatment is multidisciplinary and standard chemotherapy may benefit from adjuvant treatments such as PDT. The application of photodynamic action on post-chemotherapeutic tissue *in vitro* shows additional cellular damage as determined by histological analysis. This work presents preclinical studies on an *in vitro* tissue model of Rose Bengal PDT, which has potential for clinical treatment of breast cancer. This study has demonstrated the importance of an analysis of histopathological characteristics during breast cancer treatment. The effects of *in vitro* photodynamic action applied in this study suggests that the application of PDT after chemotherapy can aid in breast cancer cell eradication.

## Figures and Tables

**Table 1:** Rose Bengal concentrations applied to breast cancer tissue samples.

Rose Bengal Concentration Values
0.02 g/mL used for 5 samples after chemotherapy
0.03 g/mL used for 5 samples after chemotherapy
0.05 g/mL used for 5 samples after chemotherapy

**Table 2:** The effectiveness of PDT.

**% of dead cells**	**Rose Bengal Concentration**		
	0.02 g/mL *	0.03 g/mL *	0.05 g/mL *
	32 ± 2%	46 ± 3%	93 ± 3%

* experiment performed on five post-operative breast cancer tissues.

**Table 3:** Results of optimization of the distance between the laser light source and the sample.

The Distance between the Laser Source and Tissue [cm]	Power the 532 nm Light Dose [$\frac{J}{{\mathrm{cm}}^{2}}$]
15	9 ± 2

## Data Availability

The data presented in this study are available on request from the corresponding author.
